# Implementing six multi-month dispensing of antiretroviral therapy in Ethiopia: perspectives of clients and healthcare workers

**DOI:** 10.1186/s12913-023-09549-7

**Published:** 2023-05-31

**Authors:** Joanne E. Mantell, Jennifer M. Zech, Tsitsi B. Masvawure, Tamrat Assefa, Mitike Molla, Laura Block, Dereje Duguma, Zenebe Yirsaw, Miriam Rabkin

**Affiliations:** 1 New York State Psychiatric Institute and Department of Psychiatry, HIV Center for Clinical and Behavioral Studies, Gender, Sexuality and Health Area, Columbia University Irving Medical Center, New York, New York United States of America; 2 ICAP at Columbia University, New York, NY United States of America; 3 Health Studies Program, Center for Interdisciplinary Studies, College of the Holy Cross, Worcester, MA United States of America; 4 ICAP Ethiopia, Addis Ababa, Ethiopia; 5 Addis Ababa University, Addis Ababa, Ethiopia; 6 Ministry of Health, Addis Ababa, Ethiopia; 7 Departments of Medicine and Epidemiology, Columbia University Irving Medical Center, New York, NY United States of America

**Keywords:** Antiretroviral therapy, Multi-month drug dispensing, HIV, Ethiopia, Mixed-methods research, Differentiated service delivery

## Abstract

**Background:**

Multi-month dispensing (MMD) of antiretroviral therapy (ART) is an integral component of differentiated HIV service delivery for people living with HIV (PLHIV). Although many countries have scaled up ART dispensing to 3-month intervals, Ethiopia was the first African country to implement six-month dispensing (6-MMD) at scale, introducing its Appointment Spacing Model (ASM) for people doing well on ART in 2017. As of June 2021, 51.4% (n = 215,101) of PLHIV on ART aged ≥ 15 years had enrolled in ASM. Since little is known about the benefits and challenges of ASM perceived by Ethiopian clients and their healthcare workers (HCWs), we explored how the ASM was being implemented in Ethiopia’s Oromia region in September 2019.

**Methods:**

Using a parallel convergent mixed-methods study design, we conducted 6 focus groups with ASM-eligible enrolled clients, 6 with ASM-eligible non-enrolled clients, and 22 in-depth interviews with HCWs. Data were audio-recorded, transcribed and translated into English. We used thematic analysis, initially coding deductively, followed by inductive coding of themes that emerged from the data, and compared the perspectives of ASM-enrolled and non-enrolled clients and their HCWs.

**Results:**

Participants enrolled in ASM and HCWs perceived client-level ASM benefits to include time and cost-savings, fewer work disruptions, reduced stigma due to fewer clinic visits, better medication adherence and improved overall health. Perceived health system-level benefits included improved quality of care, decongested facilities, reduced provider workloads, and improved record-keeping. Although non-enrolled participants anticipated many of the same benefits, their reasons for non-enrollment included medication storage challenges, concerns over less frequent health monitoring, and increased stress due to the large quantities of medicines dispensed. Enrolled participants and HCWs identified similar challenges, including client misunderstandings about ASM and initial ART stock-outs.

**Conclusions:**

ASM with 6-MMD was perceived to have marked benefits for clients and health systems. Clients enrolled in the ASM and their HCWs had positive experiences with the model, including perceived improvements in efficiency, quality and convenience of HIV treatment services. The concerns of non-ASM enrolled participants suggest the need for enhanced client education about the model and more discreet and efficiently packaged ART and highlight that ASM is not ideal for all clients.

## Introduction

Differentiated service delivery (DSD) is a tailored approach to HIV program design that increases the diversity and flexibility of service delivery models for the increasing numbers of people living with HIV (PLHIV) on antiretroviral therapy (ART) [1]. By adjusting the location and frequency of HIV services and expanding the cadres of healthcare workers (HCW) providing services for PLHIV, DSD can improve the coverage, quality, efficiency and impact of HIV programs as well as patient satisfaction [2, 3] and retention in care [4–6]. Successful DSD requires careful design, implementation, and scale-up that is person-centered and grounded in evidence [7]. The selection and optimization of contextually-appropriate person-centered DSD models are priorities for many health ministries, donors, and program implementers [8].

Ethiopia has made great progress in scaling up HIV treatment, and UNAIDS estimates that 74% of the 670,000 PLHIV in Ethiopia had access to ART in 2019 [9], and by December 2022, 454,257 PLHIV were on ART [10]. As the Ethiopian Ministry of Health works to increase ART coverage, it has been implementing DSD models to improve quality, acceptability, and efficiency. The use of multi-month dispensing (MMD) and appointment spacing is prioritized for people doing well on ART to minimize missed clinic appointments, nonadherence to medication and disengagement from care due to high costs of travel, lost wages, stigma and long wait times at ART clinics [11–13]. This DSD strategy is aligned with the 2021 World Health Organization recommendation that people who are established on ART should be offered refills lasting 3–6 months, with a preference for six-monthly dispensing (6-MMD) where feasible [14]. Although many countries have shifted to 3-MMD, Ethiopia was among the first to implement 6-MMD at scale, as part of its Appointment Spacing Model (ASM) launched in July 2017. As of Fiscal Year 2021 (July 01, 2020-June 30, 2021), 215,101 PLHIV aged ≥ 15 years (51.4% of those on ART) were enrolled in ASM [15].

In Ethiopia’s conventional model of ART service delivery, new clients are seen every two weeks in their first month of ART, monthly between months two and six, and every three months for the next six months (months 7 to 12). Clients who are doing well are then eligible to enroll in the 6-MMD ASM. Those who decline to enroll in ASM continue to be seen at the ART clinic every one to three months.

**Fig. 1:**
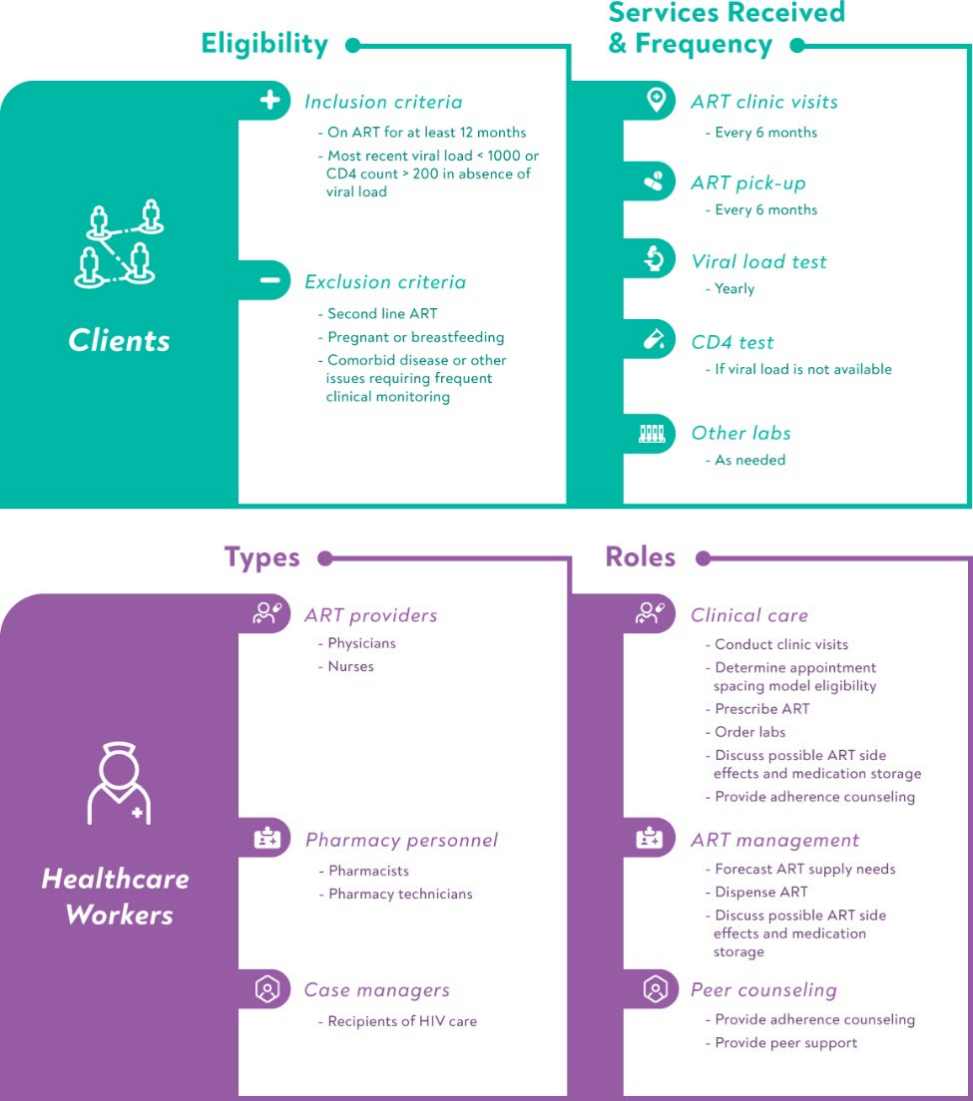
Characteristics of Ethiopia’s appointment spacing model

In contrast, ASM is a facility-based individual DSD model in which adults doing well on treatment are seen at health facilities twice a year and receive a six-month supply of ART and a clinical evaluation (see Fig. 1). Between 2017 and 2019, the six-month supply of ART was dispensed as six bottles of 30 tablets each for a total of 180 tablets; starting in 2020, clients received two bottles of 90 tablets. Eligibility criteria include having been on ART for at least one year with evidence of treatment success (e.g., suppressed viral load or rising CD4 count) and the absence of other conditions requiring more frequent clinical monitoring. Eligible clients are provided with information about the model and offered the choice to opt into the ASM. Medical officers and nurses provide clinical care, case managers -- themselves recipients of HIV treatment -- provide adherence counseling, and pharmacy staff forecast drug supply needs, dispense ART and counsel clients about possible side effects and medication storage. Although telephone follow-ups are not required, some HCWs phone clients to remind them of their clinic appointments.

There is growing evidence supporting the use of MMD in diverse DSD models, including both community-based and facility-based models for individuals and groups [16]. Emerging data suggest that MMD reduces missed appointments, as well as the costs of transportation and time spent at health facilities [17, 18]. Several trials have shown the promise of 6-MMD to support retention in HIV care and viral load suppression. A cohort study of 62,084 PLHIV in Zambia found that 6-MMD was associated with fewer missed visits, higher medication pick-ups and fewer clients lost to follow-up compared to a monthly drug refill model [6]. In Nigeria, a retrospective cohort analysis of 2,089 people on ART found that those receiving MMD were more than twice as likely to be retained on ART vs. those with monthly drug refill visits. A cluster randomized non-inferiority trial of 2,150 people on ART in South Africa found similar 12-month [19] and 24-month [20] retention rates among people in adherence clubs dispensing 6-MMD vs. those receiving more frequent medications (2-MMD, 4-MMD); at 24 months, viral load suppression was higher in the 6-MMD group. Similarly, in a cluster randomized non-inferiority trial in Zimbabwe, retention in ART care at 12 months in community ART groups using 3-MMD and 6-MMD was non-inferior to standard of care [21]. A facility-based cluster-randomized non-inferiority trial of 8,719 people at health facilities in Malawi and Zambia found that 6-MMD was non-inferior to standard of care and 3-MMD on retention in care at 12 months [22]. Other studies have reported similarly high retention in DSD models using MMD [4, 5, 23] as well as increased viral load suppression [5, 24, 25], cost-savings for clients [26], and HCW and client satisfaction [21, 27, 28].

Following a pilot phase in 2017, Ethiopia’s Ministry of Health and its partners rapidly scaled up ASM enrollment to 100,000 people (23% of those on ART) by January 2019. However, 20% of those offered ASM enrollment opted not to participate, prompting Ethiopia’s MOH to engage ICAP at Columbia University to conduct a mixed-methods study to explore the lived experiences of PLHIV and HCWs regarding MMD and ASM and why participants chose to enroll, and others declined to enroll as well as identify program successes and challenges.

## Methods

The study adhered to the Consolidated Criteria for Reporting Qualitative Research (COREQ) 32-item checklist to demonstrate transparency of reporting and the study’s methodological strength [29]. We did not involve study participants in the development of the FGD guide or review of the transcripts.

### Study design and sampling

The study was conducted in September 2019 at three high-volume urban hospitals implementing the ASM within 100 km of Addis Ababa in the Oromia Region of Ethiopia: Adama Hospital Medical College, Nekemte Hospital, and Bishoftu Hospital. By the end of September 2019, approximately 8,053 adults were enrolled in the ASM at these health facilities. We used a parallel convergent mixed-methods design, with a combination of client focus group discussions (FGDs) and HCW in-depth interviews (IDIs) to elicit their perspectives on the ASM.

Table 1 presents the screening and enrollment status of people on ART at the study sites by the end of September 2019. Of the 8,661 people eligible for ASM enrollment at our three study hospitals, 7% declined participation and remained in the conventional treatment model.

**Table 1:** Appointment spacing model coverage at Adama, Nekemte and Bishoftu Hospitals, as of September 30, 2019

Health Facility	Currently on ART	Total Screened	Total No. of All Stable Clients on ART	Total Enrolled	Declined to Enroll	Percent of Clients on ART Enrolled in ASM
Adama Hospital	7,430	6,901	4,643	4,496	147	60.5%
Bishoftu Hospital	3,698	3,206	2,628	2,219	409	60.0%
Nekemte Hospital	2,289	1,984	1,390	1,338	52	58.5%

We purposively selected clients receiving ART based on their ASM enrollment status and sex. Sample sizes were designed to attain thematic saturation [30] and thematic variations by sex and ASM enrollment status. We aimed to conduct four FGDs at each site (with 6–12 participants per FGD): two with adults on ART enrolled in the ASM (1 male; 1 female) and two with those eligible for but not enrolled in the ASM (1 male; 1 female). HCWs directly involved in implementing the ASM (at least 1 case manager, 1 pharmacy personnel, and 1 ART provider at each health facility) were selected for the IDIs.

### Recruitment and data collection procedures

Prior to data collection, study staff conducted sensitization meetings with clinical staff at each health facility to describe the study protocol and procedures. Health facility staff then identified adults on ART currently eligible for ASM and referred them to the study team. HCWs, in turn, were recruited at a study information session by study staff who explained the study, invited those eligible to participate, and obtained written informed consent.

Six Ethiopian research assistants (3 female; 3 male) who had master’s degrees or were enrolled in a master’s program and experienced in conducting qualitative and quantitative research participated in a five-day workshop to review study tools and procedures. Training focused on augmentation of qualitative research skills (e.g., listening, reflecting, summarizing), reflexivity and positionality in qualitative inquiry, and research ethics [31]. Research assistants practiced conducting FGDs and IDIs with each other as part of the training so that they would become familiar and comfortable with these tools.

All FGDs and IDIs were conducted at the health facilities. Each FGD was conducted by two research assistants in Amharic or Oromo and took approximately 90 min, whereas IDIs with HCWs lasted about one hour and were conducted in English or Amharic. Interviewers and FGD facilitators were not always matched by the sex of the participants due to language constraints.

### Domains assessed

FGD and IDI guides were developed in English, translated into Amharic and Oromo and back-translated into English to ensure accuracy. IDIs included both questions with closed-ended and open-ended responses that allowed probing in greater depth and exploration of underlying motivations, contexts and processes. IDIs focused on (1) understanding of the ASM; (2) rationale for introducing the ASM at the health facility; (3) fidelity of ASM implementation; (4) changes in the quality of services (e.g., client volume, wait time, ART adherence and missed appointments) since the introduction of ASM services; (5) thoughts about why eligible clients accept or decline the ASM; and (6) perceived barriers to and facilitators of ASM implementation. We asked similar questions of both ASM-enrolled and non-enrolled. FGD topics included (1) client knowledge, beliefs and attitudes regarding the ASM; (2) perceived benefits and challenges of the ASM; (3) reasons why clients enroll and do not enroll in the ASM; and (4) experiences of ASM-enrolled participants. Table 2 summarizes the main domains of the FGD and IDI guides and provides illustrative questions.

**Table 2:** Domains assessed in focus groups and in-depth interviews and example of questions

Domains	Examples of Questions from FGDs		Examples of Questions from IDIs
	Enrolled	Not Enrolled	
*Knowledge, beliefs, attitudes and understanding of the ASM; Fidelity of ASM implementation*	● Tell me about the ASM. How does this model differ from standard care? ● How did you learn about the ASM? Who explained it to you?	● What have you heard about the ASM for HIV treatment offered at this health facility?	● I’d like you to describe a situation in which you did not follow the ASM eligibility guidelines. Can you think of a recent situation and tell me about it?
*Benefits and challenges of the ASM; Changes in services; Perceived barriers to and facilitators of ASM enrollment*	● What is working well for you with the ASM? What benefits are you personally receiving from being in the ASM? ● Some clients report they have challenges with the ASM, whereas others do not. Have you faced any challenges? If so, could you describe those to us?	● What do you think are some of the benefits of the ASM model? ● What do you think are some of the challenges of the ASM model?	● Has the overall quality of HIV/AIDS services being offered in the facility changed compared to before the ASM was introduced?
*Reasons eligible clients offer for enrolling or not enrolling in the ASM*	● Why did you agree to enroll in the ASM? ● What do you think are some of the reasons that clients who have been offered the ASM might decide **not** to enroll?	● For those of you who have been told that you are eligible for the ASM, what are some reasons that you have **not** enrolled in the model? ● What do you think are some of the reasons that eligible clients who have been offered enrollment in the ASM opted not to participate?	● In your experience, do most eligible clients agree to be in the ASM? Why or why not? ● Do staff at this facility track eligible clients who declined ASM services in previous visits in case they have interest in ASM enrollment in the future? If so, can you tell me what strategies are in place to do this? ● Do you encourage eligible clients to enroll in the ASM? Why or why not?

### Data analysis

Audio-recordings of the FGDs and IDIs were transcribed verbatim, translated into English as needed, and uploaded into Dedoose, a web-based mixed-methods web application for qualitative and mixed-methods data analysis. Prior to analysis, bilingual senior research staff reviewed the translated transcripts for completeness and accuracy.

We used thematic analysis, initially coding data deductively based on the FGD and IDI questions, and then inductively for emergent themes or patterns of meanings [32]. Qualitative data were analyzed using an iterative process. An initial codebook was developed based on the IDI and FGD guide questions. This codebook was then entered into Dedoose, and one researcher coded the transcripts by question. Next, code reports for each question were generated and three researchers independently read these and developed a matrix of key themes per question for each cadre: HCWs and ASM enrolled and non-enrolled clients. We analyzed the FGD data by ASM enrollment status, and within the enrolled and non-enrolled groups, by sex of participants. Matrices were constructed to explore similarities and differences along these dimensions. Key themes were captured in an Excel spreadsheet. A “negotiated agreement” approach was used to reach consensus and ensure consistency in the identification and interpretation of key themes [33, 34]. The coding team also checked with Ethiopian team members about issues related to translation, linguistic nuances and cultural meanings. Analysis of code reports was a collaborative effort and allowed for exploration of different data interpretations and further analysis of transcripts until consensus was achieved, thus minimizing potential biases in interpretation.

SurveyCTO was used to capture IDI quantitative data on a tablet. Data were uploaded for storage on the SurveyCTO cloud-based system, with internal data quality checks for valid entries, skip patterns, range checks, and missing values.

### Ethical considerations

The Oromia Regional Health Bureau (Ref No. BEFO/HBTFH/1–16/1314) and the Columbia University Medical Center Institutional Review Board (Protocol IRB- AAAS2572) approved the study protocol. Participants completed written informed consent and were assured that they could decline to answer questions and/or withdraw from the study at any point. Participants were given the opportunity to ask questions. No personal identifiers were collected. FGD participants were informed that although confidentiality could not be guaranteed, all participants were asked to refrain from talking about the discussions outside of the group. FGD and IDI participants received the equivalent of 5 USD for travel expenses; FGD participants also received light refreshments.

## Results

**Fig. 2:**
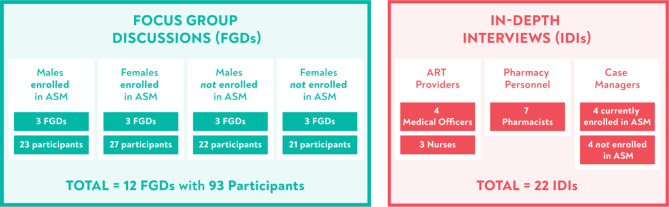
Overview of data collected

We conducted 12 FGDs, with a total of 93 PLHIV on ART and eligible for ASM enrollment (Fig. 2).

Demographic characteristics of FGD and IDI participants are described in Tables 3 and 4. FGD participants’ median age was 41.5 years (IQR 36–48), 52% were female, 20% had no formal schooling and about half had some secondary school or higher, 20% were unemployed, and 86% lived within an hour of the health facility. More than half (55%) of the FGD participants had a primary partner and among the 48 participants who knew their partner’s HIV status, most (77%) indicated that their main partner was HIV-positive. The majority (89%) had been on ART for > 5 years. By design, over half (54%) of the FGD participants were enrolled in the ASM. Of those enrolled, most (37/50) had been enrolled for more than one year.

Among the 22 IDI participants, 18 were female, median age was 39 years (IQR 34–42), 20 had worked at their health facility for at least 5 years and 17 had provided ASM services to clients for more than one year. Most ART providers (6/7) and half of the case managers (4/8) had received training on the ASM. None of the pharmacy personnel reported receiving any ASM-specific training.

**Table 3:** Demographic characteristics of focus group discussion participants

	All participants, N = 93 n (%)	Enrolled in ASM, n = 50 n (%)	Not enrolled in ASM, n = 43 n (%)
**Sex**			
Male	45 (48)	23 (46)	22 (51)
Female	48 (52)	27 (54)	21 (49)
**Age**	Median = 41	Median = 42.5 IQR = 36-48 Range = 27–65	Median = 39 IQR = 35-48 Range = 25–66
**Marital Status**			
Single (never married)	7 (8)	3 (6)	4 (9)
Married monogamous	49 (53)	26 (52)	26 (53)
Divorced	7 (8)	4 (8)	3 (7)
Separated	9 (10)	6 (12)	3 (7)
Widowed	20 (22)	11 (22)	9 (21)
Decline to answer	1 (1)	0 (0)	1 (2)
**Religion**			
Traditional	1 (1)	1 (2)	0 (0)
Roman Catholic	0 (0)	0 (0)	0 (0)
Protestant	28 (30)	13 (26)	15 (35)
Orthodox	52 (56)	29 (58)	23 (53)
Apostolic Sect	1 (1)	0 (0)	1 (2)
Muslim	11 (12)	7 (14)	4 (9)
**Residence**			
Urban	83 (89)	44 (88)	39 (91)
Rural	10 (11)	6 (12)	4 (9)
**Educational background**			
No formal schooling	19 (20)	11 (22)	8 (19)
Some primary schooling	27 (29)	14 (28)	13 (30)
Completed primary school only	0 (0)	0 (0)	0 (0)
Some secondary school	27 (29)	17 (34)	10 (23)
Completed secondary school only	12 (13)	6 (12)	6 (14)
Some tertiary schooling or higher	2 (2)	1 (2)	1 (2)
Technical	2 (2)	1 (2)	1 (2)
University	4 (4)	0 (0)	4 (9)
**Currently earning money**			
Full-time employed	23 (25)	11 (22)	12 (28)
Part-time employed	1 (1)	0 (0)	1 (2)
Self-employed	28 (30)	22 (44)	6 (14)
Self-employed, informal sector	9 (10)	4 (8)	5 (12)
Temporary jobs	13 (14)	6 (12)	7 (16)
Unemployed	19 (20)	7 (14)	12 (28)
**Money earned last year**			
<= 100 ETB	6 (6)	3 (6)	3 (7)
100–500 ETB	26 (28)	16 (32)	10 (23)
501–1000 ETB	16 (17)	10 (20)	6 (14)
1001–4000 ETB	23 (25)	10 (20)	13 (30)
> 4000 ETB	9 (10)	6 (12)	3 (7)
Don’t know	11 (12)	4 (8)	7 (16)
Decline to answer	2 (2)	1 (2)	1 (2)
**Length on ARVs**			
1-<2 year	0 (0)	0 (0)	0 (0)
2–5 years	10 (11)	2 (4)	8 (19)
> 5 years	83 (89)	48 (96)	35 (81)
**Length enrolled in ASM**			
< 3 months		2 (4)	
> 3 months, but < 1 year		11 (22)	
Over 1 year		37 (74)	
**Have a primary partner**			
Yes	51 (55)	26 (52)	25 (58)
No	42 (45)	24 (48)	18 (42)
**HIV status of primary partner**	**n = 51**	**n = 26**	**n = 25**
Negative	11 (22)	4 (15)	7 (28)
Positive	37 (74)	21 (81)	16 (64)
Partner never tested	0 (0)	0 (0)	0 (0)
Don’t know	3 (6)	1 (4)	2 (8)
**Distance live from this health facility by transport**			
Less than 10 min	7 (8)	4 (8)	3 (7)
Between 10 to 30 min	45 (48)	20 (40)	25 (58)
30 min to 1 h	28 (30)	16 (32)	12 (28)
Between 1 to 2 h	8 (9)	5 (10)	3 (7)
Over 2 h	5 (5)	5 (10)	0 (0)
**Belong to a community HIV support group**			
Yes	18 (19)	7 (14)	11 (26)
No	75 (81)	43 (86)	32 (74)

**Table 4:** Characteristics of in-depth interview participants

Total N = 22	ART Providers n = 7	Case Managers n = 8	Pharmacy Personnel n = 7
	n (%)	n (%)	n (%)
**Sex**			
Male	0 (0)	3 (38)	1 (14)
Female	7 (100)	5 (63)	6 (86)
**Age**	Median = 40 IQR = 38-47 Range = 35–50	Median = 41 IQR = 36.5-42 Range = 32–46	Median = 30 IQR = 29–35 Range = 28–46
**Professional training**			
Doctor	0 (0)		
Medical officer	3 (43)		
Nurse	4 (57)		
Pharmacist			7 (100)
**Educational background**			
Completed primary school		1 (13)	
Some secondary school		2 (25)	
Completed secondary school		5 (63)	
**Length working at this facility**			
Less than 2 years	0 (0)	0 (0)	
Between 2–5 years	0 (0)	2 (25)	
More than 5 years	7 (100)	6 (75)	
**Length working in the pharmacy at this facility**			
Less than 5 years			0 (0)
Between 5–10 years			3 (43)
More than 10 years			4 (57)
**Length personally providing ASM services in this facility**			
Less than 6 months	0 (0)	0 (0)	0 (0)
Between 6–12 months	0 (0)	2 (25)	3 (43)
More than 12 months	7 (100)	6 (75)	4 (57)
**Received training on ASM**			
Yes	6 (86)	4 (50)	0 (0)
No	1 (14)	4 (50)	7 (100)
**Length working in the field of HIV treatment**			
Less than 5 years	1 (14)		
Between 5–10 years	4 (57)		
More than 10 years	2 (29)		
**Length formally counselling clients on HIV treatment**			
Less than 5 years		0 (0)	
Between 5–10 years		4 (50)	
More than 10 years		4 (50)	

Blank space = not applicable

**Fig. 3:**
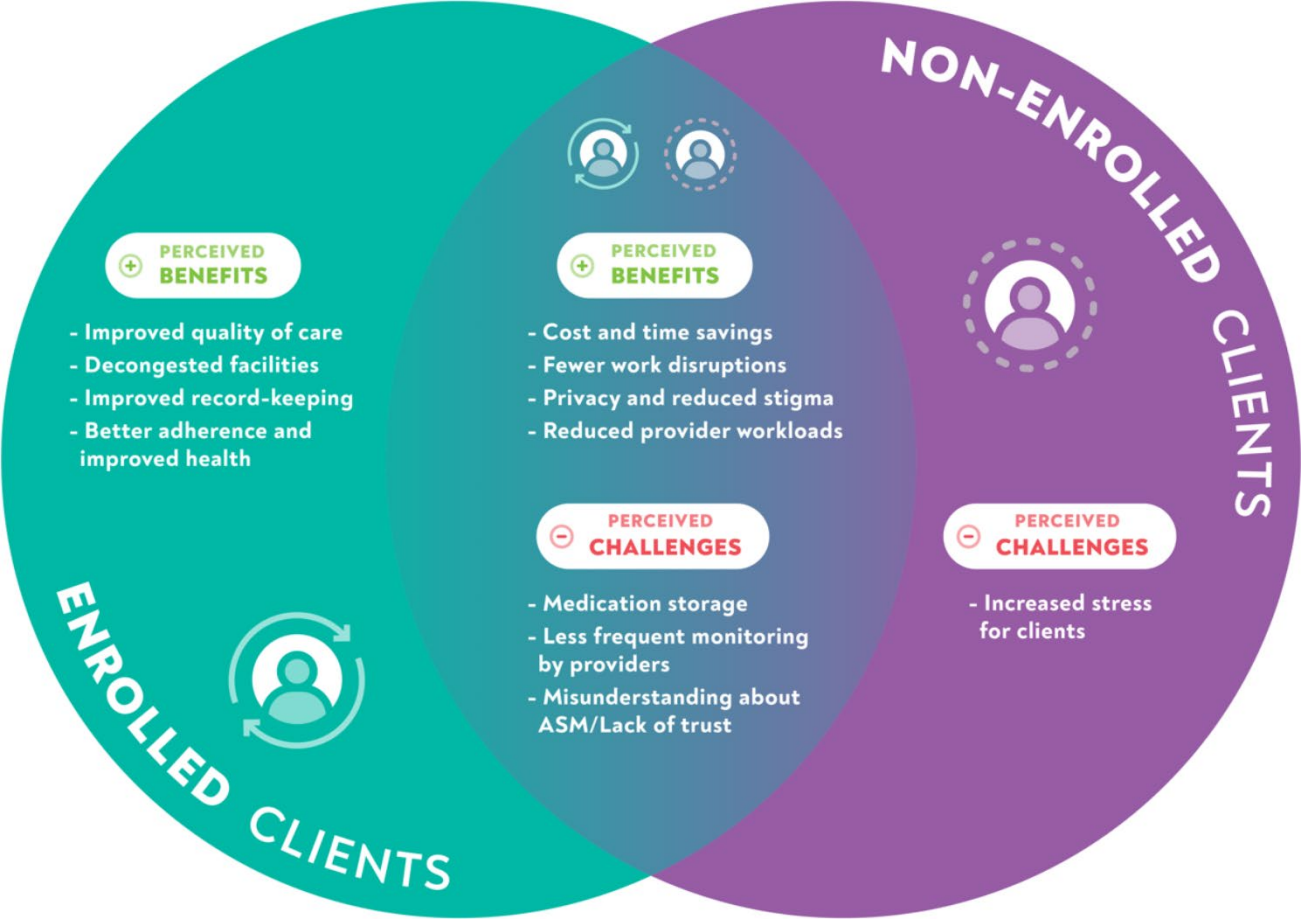
An overview of emergent themes regarding anticipated and experienced benefits and challenges among ASM-enrolled and non-enrolled clients

Below, we present the main findings, with supporting quotes, on the perceived and experienced benefits and challenges of ASM implementation for clients and health systems, categorized by key themes and ASM-enrollment status. We did not discern any gender differences in participant perspectives within and across ASM-enrolled and non-ASM enrolled FGDs. Figure 3 shows an overview of emergent key themes. Table 5 shows the frequency with which benefits and challenges emerged per theme by FGD ASM enrollment status and HCW IDIs.

**Table 5:** Participant views on benefits and challenges of the ASM

Theme		Participant Types				
		FGDs		IDIs		
		Enrolled Participants (n = 6) n (%)	Non-enrolled Participants (n = 6) n (%)	ART Providers (n = 7) n (%)	Case Managers (n = 8) n (%)	Pharmacy Personnel (n = 7) n (%)
**ASM Benefits**	Cost and time savings	5 (83)	5 (83)	4 (57)	5 (63)	5 (71)
	Fewer work disruption	3 (50)	2 (33)	1 (14)	1 (13)	1 (14)
	Privacy and reduced stigma	4 (67)	2 (33)	2 (29)	3 (38)	1 (14)
	Better adherence and improved health	4 (67)	0 (0)	2 (29)	3 (38)	1 (14)
	Improved quality of care	5 (83)	0 (0)	6 (86)	7 (86)	3 (43)
	Reduced provider workloads	2 (33)	1 (17)	5 (71)	7 (86)	6 (86)
	Decongested facilities	5 (83)	0 (0)	1 (14)	3 (38)	4 (57)
	Improved record keeping	1 (17)	0 (0)	6 (86)	2 (25)	2 (29)
**ASM Challenges**	Medication storage	3 (50)	6 (100)	3 (43)	6 (86)	4 (57)
	Less frequent monitoring by providers	2 (33)	4 (67)	2 (29)	1 (13)	1 (14)
	Increased stress for clients	0 (0)	5 (83)	0 (0)	0 (0)	0 (0)
	Misunderstanding ASM/Mistrust	1 (17)	3 (50)	0 (0)	0 (0)	0 (0)
	Medication shortage	0 (0)	0 (0)	2 (29)	6 (86)	4 (57)

### Perceived and experienced benefits of ASM

Participants described benefits to both clients and health systems. Below we describe each benefit and point out convergences and divergences across participant cadres.

### Client-level benefits

#### Time and cost-savings

Enrolled participants and HCWs noted that the ASM freed up time for clients by reducing the frequency of clinic visits, which allowed clients to attend to other obligations at home and work, including engaging in recreational activities.

*One of the benefits of this six-month medication is that it does not waste our time…there is no patient load here at the hospital. There is no long waiting time. Because of this it is very good.*

−Enrolled female FGD participant

Non-enrolled participants anticipated similar time-saving benefits of the ASM. All participant cadres highlighted the cost-savings of the ASM for clients. Enrolled participants reported reduced transportation and food expenses while at the health facility because of fewer visits.

*When they told me that I can collect every six months, I get very happy. Because first it reduces transportation costs for me, it costs 4 Ethiopian birr* [equivalent of USD 0.13] *to come here… and I pay only once every six months.*

−Enrolled female FGD participant

This benefit was also echoed by HCWs and non-enrolled participants.

#### Fewer disruptions to work schedules

Another benefit highlighted by participants across all groups was that the ASM entailed fewer disruptions to work schedules because clients did not have to take time off work every month to visit health facilities. Enrolled participants expressed discomfort at frequently requesting time off work as this could upset employers, suggest a lack of commitment to one’s job, and potentially lead to employment termination or loss of wages.

*I am working as a daily laborer…It was difficult for me to ask permission to come here every month from my employer. They don’t know where I am going…. They become suspicious and may think that I am requesting permission to avoid my work...So, the current six months-based refilling is good for me*.

−Enrolled male FGD participant

A case manager noted that non-ASM enrolled clients often requested shorter counseling sessions as they needed to rush back to their jobs.

*The other issue is about the time, especially for those who are an employee of some organization. Even during counseling, they request us to shorten the counseling time, as they have to go back to their office. In addition, requesting again and again for permission from their organization* [leads] *to frustration….*

−Case Manager IDI participant

Non-enrolled participants also perceived potential work-related benefits of the ASM especially for employees who may not have flexible work schedules that permit them to leave work for health reasons.

*It’s also* [an] *advantage for government or private employees because they may not get a leave from work as they wish.*

−Non-enrolled female FGD participant

#### Privacy and reduced stigma

All participant cadres mentioned reduced stigma as another ASM benefit. For ASM-enrolled clients, fewer appointments meant fewer opportunities to be seen, and hence potentially stigmatized, by others for seeking HIV services. This ASM benefit had a ripple effect in that enrolled clients no longer had to explain to neighbors why they visited the health facility so frequently.

*When we used to come here every month, we see different people and when we started coming every three months it was also different people that you run into. And now when you come here every six months you don’t see a lot of people. It is fewer people you run into…we see less people that discriminate against us.*

−Enrolled male FGD participant

Non-enrolled participants and HCWs also noted that the ASM was especially advantageous for clients who had not disclosed their HIV status to others as fewer health facility visits reduced the chances of being outed.

*It’s also advantageous for patients who do not want to be seen often… There are some of us who take the medicine discreetly. We don’t want our peer workers to know about our disease*.

−Non-enrolled male FGD participant

#### Improved adherence and overall health

Enrolled participants and HCWs noted that ASM ensured that clients had a stable supply of medicines between appointments, which helped to reduce the number of missed appointments or skipped doses of ART. Almost all HCWs (82%, 18/22) noted that ART adherence among ASM-enrolled clients had improved due to this stable supply. Some enrolled participants attributed their prior missed doses to transportation difficulties, challenges obtaining time off work, and medications running out between appointments. Some ASM-enrolled participants stated they were enjoying great peace of mind to the extent that they sometimes forgot about their HIV infection because they no longer worried about keeping appointments.

*When I enter the six-month medication modality, my brain… previously I was too stressful but now, I forgot it totally, I am healthy psychologically and I start focusing on my work. I start to laugh…I started even forgetting that the illness is in my body.*

−Enrolled female FGD participant.

They also highlighted that their physical and mental health had improved, including weight gain and better sleep and appetite. One ASM-enrolled female participant expressed her joy and feeling of privilege at being invited to participate in ASM: “I felt like I won the lottery”. Some enrolled participants felt that being selected to participate in ASM instilled confidence and enhanced commitment to medication adherence.

*It makes me more confident with myself because I was chosen to be part of this program. I was chosen because I was taking my medication properly. And this has motivated me to keep taking my medication on time and without fail until the end.*

−Enrolled male FGD participant

Non-enrolled participants did not perceive this benefit and tended to worry that fewer appointments associated with ASM might lead to poor adherence.

### Health system benefits

Participants felt that ASM markedly improved the quality of care and services, decongested health facilities, reduced HCW workloads and improved record-keeping. Non-enrolled participants mentioned reduced HCW workloads as the only benefit of ASM. This could be due in part to their not being asked directly about how the quality of care had improved since ASM was introduced.

#### Improved quality of care

HCWs and enrolled clients reported that the ASM had decreased workloads for health staff, thus enabling HCWs to provide higher quality services “without being in a hurry”, give better treatment and service, and not suffer job burnout.

*The more clients I enroll* [into ASM], *the more time the doctors will have with their other less stable patients to investigate properly, to take patient history and to get physical examination. Unnecessary frequent visits of the stable patients will take time from the more needy patients*.

−ART Provider IDI participant

Decreased workloads meant that HCWs could have in-depth discussions with clients about other health issues, such as safer sex, TB prevention and pregnancy, devote extra time to clients who needed extra support, correct misperceptions about ART (e.g., not taking their medication during fasting periods), counsel patients about the shelf-life of ART and advise them to keep their medication in ventilated areas away from sunlight and the reach of children.

Similar views were echoed by ASM-enrolled participants who noted that HCWs were less stressed, more attentive and cordial in their interactions with clients since the ASM was introduced. Consultations with HCWs were also now longer and more personalized.

*We used to have little time with a doctor before because there were a lot of people here and the queue was long…It was really hard when we were taking the three-month and one-month medication before. But now with the six-month medication, the doctors themselves treat us with love and ask us how we are when they see us. They even call us by our names now, which shows love from the doctors to us… Now that they are not as busy and stressed, it is good for us because we get more time with them*.

−Enrolled male FGD participant

Non-enrolled participants were not asked directly about changes in quality of care and did not mention improved quality of care as a potential benefit of the ASM.

#### Decongested health facilities

HCWs and enrolled participants reported dramatic decreases in client volumes since the ASM introduction, with some health facilities seeing as much as 50% fewer clients. Participants further stated that health facilities were cleaner and quieter as a result.

*I have seen a huge change on the cleanliness of the hospital…They have also cleaned up the building now and it looks really good. It was very chaotic and crowded when we used to come every three months, with many sick people but now it’s less crowded so it has helped with that.*

−Enrolled male FGD participant

[The] *ART pharmacy used to see 180 and 190 patients* [per day]. *They* [pharmacy staff] *all very happy because when we give for 6 months. It’s a great deal for us to decrease 60, 70 prescriptions…can you imagine how big it is to have 100 patients less?*

−Pharmacy Personnel IDI participant

Non-enrolled participants were asked about potential benefits of the ASM, but they did not mention decongested facilities.

#### Reduced HCW workload

Twenty-one of the 22 HCWs reported that they felt less rushed in their interactions with clients since the ASM rollout because their workload had decreased, mostly due to reduced client volumes. Some ASM-enrolled and non-enrolled participants also highlighted less overworked HCWs as a benefit of the ASM.

*The health institution benefits because they used to work 12 hours a day as the patient number was too high. But now because the number of patients is decreasing, they might only work half day. We also get fast treatment and take the medicine and finish what we came here to do.*

−Enrolled female FGD participant

#### Improved record-keeping

The ASM also contributed to overall efficiencies in the healthcare system, such as improvements in record-keeping and general administrative work. Pharmacy personnel in all health facilities noted reduced paperwork due to the ASM, and case managers and ART providers explained that record-keeping had improved as clients’ test results were now being retained by the health facility and attached to clients’ files for future reference, and documentation of CD4 counts and viral load results was done more consistently.

*Previously, we were concerned about covering the quota allocated to us. There were missing details in patients’ folders. This was because we were more concerned in reaching out to the thirty or forty cases allocated to each of us. But now, after the introduction of ASM, patients come once in every month. This has reduced our burden. Our case load has been reduced. Therefore, quality of filling in necessary information in the folder of patients is now good*.

−ART Provider IDI participant

Case managers further noted that clients now received laboratory services quicker.

*There is improvement/change now. If any person gets sick and comes, his history is attached to his card and kept…. During the earlier times, you bring your result and you are told to take your result with you. That means, you get treated and throw away your result. But now there is no such practice. Now your result is attached to your file… So, when you come next time, what was the diagnosis last time, your current status, the doctor/the physician* [can] *see and tell you the change*.

−Case Manager IDI participant

ASM-enrolled clients reported that the ASM had resulted in fewer patient cards or files getting misplaced or lost, thereby ensuring a more efficient service.

*…We used to really suffer because our cards went missing all the time and now that is not the case. That has changed now. I haven’t had that issue since I started the six months medication.*

−Enrolled male FGD participant

## Perceived and experienced challenges of ASM

Participants were also asked about the challenges of the ASM. Below we report on challenges that emerged, most of which were at the client and health system levels only. Most of the challenges reported reflected anticipated concerns that non-enrolled participants had about the ASM compared to actual challenges experienced by the enrolled.

### Client-level challenges

#### Medication storage, privacy and safety concerns due to pill volumes

Proper and safe storage of ART medications was voiced by clients and HCWs as a real or potential challenge of the ASM. Non-enrolled ASM participants indicated they did not have space to store a six-month supply of pills safely or privately, hence their reluctance to enroll in the ASM. For some, the increased number of pills attendant with a six-month supply was an issue as they feared that other household members would find the stored medications, thus leading to unwanted disclosure.

*I do not want people to see it* [medicines] *so I just shove it somewhere to hide it from being seen and I just pull one out, hide it and take it to work with me. So … that is the reason why I do not want to take the six months one….*

−Non-enrolled male FGD participant

*The other reason is they fear that other people might notice when they take that much medication because sometimes … some people live in the same house and don’t want the others to find out about their situation. They fear that the people living with them might find the medicine if they put that much medicine in their home.*

−Case Manager IDI participant

Keeping the medications out of the reach of children was a related concern. Non-enrolled and some enrolled participants thought that a six-month supply would make it difficult to store medicines safely and away from children because of the sheer volumes involved and the limited space in many homes.

*We were told to take the* [six-month] *medicine, but we were not happy… most of us are living either in leased houses or in a single room in which our children could have easy access and may play with it* [medicines]. *Once, I found my children playing with it.*

−Non-enrolled female FGD participant

*I am telling you this so that you can understand why I want to go back to the three-month medication. The reason for me is storage…because I am having issues with where to store the medicine since I have kids in the house.*

−Enrolled female FGD participant.

Medications that were not stored securely could be taken by others. An enrolled participant shared an experience in which his mother pilfered his medications after he stored them in a place that was easily accessible to others in his household.

*Let me tell you something that happened. My mother used to live with me before and she used to think my medication was good so she would take some and hide it then take it!…Yes, she was swallowing it*.

−Enrolled male FGD participant

Large pill volumes also made it difficult for clients to transport the medication discreetly from health facilities to their residences. HCWs noted that some clients discarded the ART packaging to prevent the medicines from being seen by others at health facilities and to make it easier to transport the medicines inconspicuously. They also expressed concern about the ART medication losing its potency because of improper storage.

*… some people want to hide that they have the disease. thus hiding this huge number of medicine is difficult for them and I was observing from their faces worry about the visibility of the medication to others when they collect it from the pharmacy*.

−Case Manager IDI participant.

Non-enrolled and some enrolled participants noted that medicines would spoil in hot weather due to the absence of refrigerators or to water damage in flood-prone areas as potential challenges of the ASM.

… *The temperature of my house is also very hot because the ceiling is too* [low] *and the house is very small… it’s very narrow and confined. So, I have to be careful by putting the medicine in a cold and dry place.… It’s just that my house is very hot and that constrains me from taking the medicine every 6 months.*

−Non-enrolled female FGD participant

#### Less frequent monitoring and support

Non-enrolled clients noted the reduced number of health facility visits as another reason for not enrolling in the ASM. They explained that they preferred frequent visits as these facilitated social bonding with HCWs and other clients. Participants described some HCWs as nurturing and supportive, hence less frequent health facility visits were seen as depriving them of much needed informational and psychosocial support. The health facility was also seen as a place where clients could acquire new information about other health issues, get their questions answered, and learn from the experiences of others. For some non-ASM enrolled clients, frequent check-ups reassured them that they were doing well, and they worried that reduced facility visits could lead to complacency, forgetfulness to take ART, loss to follow-up, and impaired health.

*I did not want to take it because it would loosen* [weaken] *my relationship with the doctors, and those I share common problems with. I would lose these people. Six months without interaction with them means like being dead to me. That was why I left it. When I come here monthly or once in two months, my people here would tell me about the good changes they have seen in my appearance. If they observed positive changes on my face, they would say: ‘Oh, how beautiful you have become! That would make me very happy’*.

−Non-enrolled female FGD participant

*There is insufficiency in counseling. There is a difference in counseling. Previously we come in the morning and take counseling/education. The service was after the counseling. Now it is after six months. It means you will not get counseling for five months. They don’t facilitate the schedule for counseling and call us.*

−Enrolled male FGD participant

Some HCWs reported initially being concerned that enrolled clients might not take their medications properly when the ASM program was first introduced at their health facility. They therefore checked on their clients regularly by telephone, assessing adherence and reminding them that taking ART did not mean that they are “off the hook or that they are healed” and stressing that adherence was essential. However, this initial skepticism was proved wrong when they saw that adherence and viral suppression levels did not fall among ASM-enrolled clients. Some enrolled participants noted that some PLHIV lacked confidence in their ability to adhere to a six-month ART medication pick-up schedule and thus needed to interact with the health facility frequently.

*There are also other people, other patients that worship health centers and like to be here all the time and for them what is important is seeing the doctor’s face and talking with their doctor than getting the medication. That is what I believe.*

−Enrolled male FGD participant

#### Increased stress for clients

A potential challenge mentioned only by non-enrolled participants was that they would find it stressful and frustrating to have a six-month ART supply in their homes because it would be a constant visual reminder that they were sick.

*It is frustrating. You would see the large quantity of medications whenever you are around. You would ask yourself when you would be able to finish all those drugs.*

−Non-enrolled female FGD participant

Another non-enrolled participant reported being very stressed when her husband was enrolled in the ASM because of the large number of pills he was given, and the amount of monitoring it took to ensure that he took the medications correctly. She gave this as her reason for not enrolling in the ASM.

*I did not like it when my husband used it. I hated it when he brought all those drugs home…the quantity would make me scared… Its large quantity would make people depressed…Oh, my husband! When he used to take the six months’ medication, I used to look after him like a child so that he would take his medication properly*.

−Non-enrolled female FGD participant

Several non-enrolled participants believed that the ASM placed an unnecessary burden on the client for maintaining ART adherence over a six-month period.

#### Misunderstandings about ASM and mistrust in the healthcare system

Non-enrolled participants were aware of the eligibility criteria for ASM enrollment but described concerns that suggested they neither understood how the ASM operates nor trusted the healthcare system. Unlike enrolled clients, some non-enrolled clients thought they would be limited to twice-yearly health facility visits and would not be welcomed if they wanted more frequent visits, that the conventional ART pills differed from the regimen given at six-month intervals with the latter being less effective, and/or that the six-month medication refills would expire prior to their next scheduled health facility visit.

*Personally, I prefer the three months’ medication. This is because one can change the medication quickly if it happens to have side effects. But the longer the duration, the higher the risks. That means we would continue to take the medication for six months despite its side effects, for we would believe in its effectiveness*.

−Non-enrolled female FGD participant

HCWs explained that they counseled clients about the importance of coming to the health facility before their six-month appointment if they were experiencing any problems. Enrolled participants also offered reasons why some ASM-eligible clients could misunderstand ASM services.

*… if a person says no to take the six-month medication after being advised by the health workers, maybe that person does not have a good impression or does not trust the health workers.…if I believe you are here to give me help and think of nothing but what is best for me as a patient, then I would have to trust your advice and take the medication you recommend for me.*

−Enrolled male FGD participant

Referring to a client who refused to enroll in the ASM, an enrolled participant noted that concern about expiration of a six-month medication supply was a deterrent to ASM enrollment.

*The person told me that the medicine might expire if we took it once every 6 months. So that person decided to come here to take the medicine every month.*

−Enrolled female FGD participant

### Health System-Level challenges

#### Medication shortages

Initial shortages of ART when ASM was first introduced was another area of discontent for clients and pharmacy personnel. Although this problem had been resolved at the time of data collection, some enrolled participants still expressed concern about medication shortages in the future.

## Discussion

This is one of the first studies to explore the perspectives of clients and HCWs on Ethiopia’s 6-MMD ASM. Our findings add to the growing literature on DSD and MMD and suggest ways to optimize ASM implementation. In our study, ASM clients and HCWs were enthusiastic about the reduced frequency of health facility visits, noting numerous benefits for clients, such as saving costs and time, minimizing disruptions to work schedules, increasing privacy, and improving medication adherence and physical and mental health. Offering enrollment in ASM was a sign of treatment success which validated clients’ current medication practices and reinforced their commitment to medication adherence. At the health system-level, participants noted improved service quality, decongested health facilities, reduced HCW workloads and improved record-keeping as the key benefits of the ASM. The main challenges of the ASM perceived at the client-level were concerns about ART storage at home, reduced interactions with and support from HCWs, increased stress due to large pill volumes and misunderstandings about the ASM among those not enrolled in the model. ART stock shortages experienced in the early days of ASM implementation were the only challenges noted at the health-system level.

A key finding of our study is that the ASM has the potential to improve client perceptions of the quality of services they receive at health facilities, thus leading to better healthcare engagement. Enrolled clients and HCWs felt that the quality of healthcare services overall had improved under the ASM and they described health facilities as being less hectic, more organized and pleasant to be around since the ASM was introduced. HCWs acknowledged that they were less stressed and had time for longer consultations with clients because the ASM had helped to decongest health facilities and reduce client volumes. Enrolled clients felt that HCWs were more attentive and more caring. Many studies have shown that the organization of a health facility’s services, its environment, and HCWs’ attitudes and responsiveness can either facilitate or hinder healthcare utilization by community members [34–37]. HCWs are “partners and agents in the process of healthcare” [38].

Our study findings echo those reported in other studies regarding the relationship between perceptions of healthcare quality and healthcare utilization, such as quality of care being lowest where patient volume was highest [39] and clients rating cleanliness and tidiness as important determinants of health facility use [35]. A Malawi study found that six-month ART dispensing resulted in a reduction in workload due to clinic decongestion resulting from fewer medication refill visits [27]. Work environment conditions, such as high patient volumes and heavy workload, are a major source of stress for HCWs and have been shown to affect their work performance and the quality of care provided to patients [40]. Additionally, poor attitudes and practices among HCWs, such as disrespect, has impeded healthcare utilization [41]. Although non-enrolled participants in our study did not mention decongested facilities and enhanced quality care as ASM benefits, we believe that the ASM has the potential to improve the quality of care for all clients, not just those enrolled in the program. ASM had other operational efficiency benefits, such as improving documentation of client data and contributing to fewer client records being misplaced or lost. Study findings further suggest that policymakers and program developers in Ethiopia should regularly monitor work-related stress of ART providers and develop strategies to promote HCWs’ well-being and preclude burnout.

Our study also indicates that the ASM has the potential to reduce clients’ concerns about HIV stigma. For some ASM-enrolled clients, fewer visits to health facilities restored a sense of normalcy and enabled them to focus on other aspects of their lives rather than on their identity as a person living with HIV. Additionally, enrolled participants reported that the ASM had reduced their stress levels because they no longer worried about keeping appointments or negotiating frequent time off work, which could raise employers’ and co-workers’ suspicion that they were HIV-positive. This sense of normalcy has been noted among clients in studies conducted in South Africa [42] and Malawi [27]. Preference for reduced clinic visit frequency has been reported in other studies [21, 28, 43].

Non-enrolled clients in our study expressed concerns about unwanted disclosure of their HIV status, which highlights the persistence of HIV-related stigma in Ethiopia [44, 45] and a dearth of mutual support platforms in communities. Recent UNAIDS reports have highlighted stigma as a real and pernicious threat to HIV control efforts as it can deter individuals from accessing HIV services [46] and lead to poor treatment outcomes and suboptimal adherence [47]. The presence of perceived stigma in our study is aligned with several other studies in Ethiopia, which report lower likelihood of enrolling in ASM [48] and increased odds of psychological distress [44] among individuals who report experiencing any form of stigma. Models such as the ASM that entail fewer health facility visits can play a vital role in reducing HIV stigma for PLHIV.

In terms of potential challenges of the ASM, some non-enrolled clients highlighted lack of suitably private places to store large volumes of medications discreetly, out of sight from other household members and neighbors, as reasons for not enrolling in the ASM. A South African study reported similar privacy concerns among ART clients who believed that carrying a six-month ART supply back to their homes using public transportation would lead to unwanted disclosure of their HIV status due to noise of the pill boxes rattling against each other [42]. However, none of the clients in the 3-MMD and 6-MMD arms of a Malawian study noted medication storage as problematic in contrast to half the HCWs in our study who were concerned about medication storage in clients’ homes [27]. In a qualitative study of PLHIV in Tanzania, participants identified three attributes of ART packaging -- visual identification, bulkiness, and clattering of pills in the ART bottles -- that fostered opportunities for stigma and motivated self-repackaging [49].

Discreet ART packaging that minimizes the number of pill bottles is a simple intervention that can enable an individual to circumvent being identified as a PLHIV. Ethiopia has moved from 30-tablet to 90-tablet bottles for 6-MMD. Pharmaceutical companies should consider ways to package ART inconspicuously that will preclude inadvertent disclosure of HIV status, e.g., small blister packs. Community-based peer support systems should also be developed to supplement the support received in health facilities in ways that do not exacerbate stigma and that protect the confidentiality of those who opt for the ASM.

Medication storage concerns provided important insights into the housing challenges faced by participants in our study, such as unstable and insecure accommodations, which required them to move frequently or share a few rooms with many other people, thus affording them little privacy. Lack of suitable storage due to participants’ living conditions also stoked fears about children finding and ingesting the medication. Ethiopia’s weather posed additional storage concerns as participants worried about the effects of heat and water on the medication. Studies in other countries have reported similar storage concerns [27, 28] A positive aspect, however, was that participants in our study acknowledged that disclosing their HIV status to household members could alleviate their concerns about storage as hiding their ARV pills would no longer be necessary. Storage concerns are not universal, however, as illustrated by a qualitative study in Khayelitsha, South Africa, which found that storing a six-month supply of ART refills was not problematic for clients in adherence clubs [42]. However, in our study, given that medication storage was a concern of both clients and HCWs, this should be addressed head-on during the orientation of newly-enrolled clients and coupled with on-going treatment literacy education on how to keep medications safely and mitigate the potential for HIV stigma. The U.S. President’s Emergency Plan for AIDS Relief (PEPFAR) Country Operational Plan (Fiscal Year 2021) recommended the identification of safe storage requirements for larger pack sizes with MMD [50].

Another potential challenge of the ASM was limited access to counseling. This emerged as an issue for non-enrolled clients, a number of whom were worried that the ASM would reduce opportunities for facility-based counseling and support, especially for clients who are dependent on supportive feedback from HCWs. Participants in our study held their HCWs in high regard and trusted their advice and looked forward to regular interactions with HCWs who motivated them to continue their treatment regimen [51]. Thus, some were concerned that appointment spacing could disrupt the close relationships between clients and HCWs. Given that the majority of our study participants had been on ART treatment for five years or more, comfort with their existing/routine schedule of clinic visits and medication pick-up may have deterred some eligible participants from shifting to a longer interval between clinic visits.

Lack of HCW support due to reduced clinical visits has been noted in several other DSD model studies. In Uganda, stable clients enrolled in community-based models who did not have a clinical need for monthly visits not only voiced concern about the loss of support gleaned from regular face-to-face interactions with their HCWs, but also felt detached from the healthcare system and believed that longer intervals between health facility visits would limit their ability to receive comprehensive care [52]. Fear of disengagement from the healthcare system due to infrequent meetings with their HCWs and concern about inability to receive comprehensive care have also been reported among clients enrolled in community-based DSD models. These concerns also point to limited understanding of the model among non-enrolled clients in our study as some did not realize that they could still visit the health facility between appointments if they experienced a health problem.

As the ASM expansion continues, it will be important to assure clients that they can continue to receive counseling services between appointments as this was a barrier to ASM enrollment for some non-enrolled participants in our study. Health facilities are also social spaces that allow for vibrant social exchanges and social support among clients. This social hub provides a respite from routine daily activities. Some non-enrolled participants equated reduced frequency of clinic visits with loss of social capital as they would no longer regularly see other clients with whom they socialized. Access to community-based counseling and psychosocial support services could help to bridge the gap between visits to health facilities. Client support groups at ART health facilities should be considered as concerns about community stigma could be a barrier to community-based support groups.

In the spirit of DSD, HCWs need to support clients who are unwilling to enroll in ASM for whatever reason. Also, HCWs should continue conversations about ASM, reinforce its benefits with these clients at follow-up visits, and assess whether their views on ASM have changed. The strength of DSD is its recognition that client-centered care entails listening to and prioritizing client interests and preferences, with the aim of improving health outcomes. Clients should not be pressured to enroll in the ASM if they do not want to do so, even after being counseled on the benefits of the model.

## Strengths and limitations

This is one of the first studies focused on implementation of appointment spacing and 6-MMD in a country that has scaled up this differentiated ART service delivery model. A main strength of the study was eliciting the perspectives of clients enrolled in ASM and a comparison group of those who were eligible but opted not to enroll in the program. Asking similar questions to both groups of participants provided rigor to the study and enhanced understanding of motivations to participate or not in this model. Drawing on the views of multiple stakeholders – clients and HCWs – facilitated triangulation of data and validity of data interpretation. Our study also had a number of limitations. First, we conducted the study in three health facilities in one region of the country, Oromia, and findings may not be generalizable to ART clients and HCWs elsewhere in Ethiopia. However, this national model was intended to be implemented in the same way and therefore should be broadly representative of the ASM throughout Ethiopia. Second, our client sample consisted of those with suppressed viral load (or high CD4 count) and may not reflect the experiences of people on ART who have not achieved viral suppression. Four-fifths of the non-enrolled clients were treatment-experienced, which may have led some to resist changing the frequency of their clinic visits and ART refills. Study findings may not apply to clients who have been on treatment for less time. Finally, in some sites, interviewers and FGD facilitators were not matched by participants’ sex due to language constraints. However, evidence that matching demographic characteristics of interviewers and interviewees improves data validity is sparse [53], but some studies suggest that gender context in FGDs, depending on discussion topic and whether the groups are gender-mixed, can affect conversation dynamics, e.g., inhibiting comfort in discussing health-related topics [54]. We did not discern any differences in the narratives of female and male participants.

## The way forward

Clients’ and HCWs’ experiences with the ASM provide important data to inform the Ethiopian MOH about how the model is unfolding and attendant implementation facilitators and roadblocks. The ASM with MMD offers a streamlined and efficient way to provide client-centered treatment and engage clients in HIV care. Also, as several researchers recently noted, the generally positive experiences of clients and HCWs in 6-MMD models provides important information for countries that have not initiated but may be contemplating introduction of this DSD model into their ART service programs [52, 55, 56].

Additional research is needed to explore whether appointment spacing and 6-MMD may be effective models for clients who are not established on ART, e.g., those who have difficulty attending appointments and obtaining medication refills. Studies of appointment spacing with longer intervals than six months, e.g., yearly, are needed to determine if this efficiency will also yield optimal continuity of HIV treatment and adherence and improved viral suppression. Moreover, robust quantitative studies to assess the impact of the ASM on continuity of HIV treatment, ART adherence, viral load suppression and cost-effectiveness are a priority, as are comparisons of the effectiveness of the ASM with other facility- and community-based DSD models.

In 2020, the COVID-19 pandemic led to the expansion of MMD throughout sub-Saharan Africa [57, 58], and to key changes in Ethiopia’s DSD models. Ethiopia’s Ministry of Health made several adaptations to maintain continuity of ART services: (1) 6-MMD for ART clients eligible for ASM, including those who previously refused/declined ASM; (2) 3-MMD for “unstable” clients with high viral loads if they are receiving enhanced adherence counseling as well as for children, adolescents and pregnant women; (3) fast-track ART refills; (4) family-based refills for PLHIV with other co-morbidities and/or if over 60 years of age, have family members who can pick-up their ART; and (5) home ART delivery [59]. The ASM still accounts for the largest proportion of PLHIV enrolled in a DSD model in Ethiopia. Of note, the proportion of eligible clients enrolled in ASM at the three health facilities has continued to increase, from 59 to 61% in September 2019 at the time of our study, to 74% as of July 2021 [60].

Long-acting ART injectables, e.g., cabotegravir + rilpivirine, approved by the US Food and Drug Administration in January 2021 [61], have been found to maintain viral suppression in adults for two years and will likely help to overcome pill-related stigma and storage issues. Although not currently available in Ethiopia, once recommended by the World Health Organization the Ministry of Health is likely adopt this treatment modality as an option for PLHIV [62], and potentially improve adherence and retention in HIV care.

## Conclusions

Clients enrolled in the ASM and their HCWs had strongly positive experiences with the model, which they perceived to improve the efficiency, quality and convenience of HIV treatment services. Clients not enrolled in the ASM had more concerns about appointment spacing and 6-MMD. These variations in clients’ perspectives support the observation that no one DSD model will be acceptable to all clients and highlight the need for client choice and diverse models of care.

## Data Availability

All data are fully available without restriction on this FigShare database. Also, available from the corresponding author on reasonable request. *Conflicts of interest/Competing interests*. The authors have declared that no competing interests exist.

## Abbreviations

ART: Antiretroviral therapy
ASM: Appointment Spacing Model
DSD: Differentiated Service Delivery
FGD: Focus Group Discussion
HCW: Healthcare Worker
IDI: In-depth Interview
MMD: Multi-month Dispensing
PLHIV: People Living with HIV
